# Memory Category Fluency, Memory Specificity, and the Fading Affect Bias for Positive and Negative Autobiographical Events: Performance on a Good Day–Bad Day Task in Healthy and Depressed Individuals

**DOI:** 10.1037/xge0000617

**Published:** 2019-06-13

**Authors:** Caitlin Hitchcock, Jill Newby, Emma Timm, Rachel M. Howard, Ann-Marie Golden, Willem Kuyken, Tim Dalgleish

**Affiliations:** 1 MRC Cognition and Brain Sciences Unit, University of Cambridge; 2 Department of Psychiatry, University of Oxford; 3 MRC Cognition and Brain Sciences Unit, University of Cambridge, and Cambridgeshire and Peterborough NHS Foundation Trust

**Keywords:** autobiographical memory, positive bias, mental health, depression

## Abstract

In mentally healthy individuals, autobiographical memory is typically biased toward positive events, which may help to maintain psychological well-being. Our aim was to assess a range of important positive memory biases in the mentally healthy and explore the possibility that these biases are mitigated in those with mental health problems. We administered a novel recall paradigm that required recollection of multiple good and bad past events (the Good Day–Bad Day task) to healthy and depressed individuals. This allowed us to explore differences in memory category fluency (i.e., the ability to generate integrated sets of associated events) for positive and negative memories, along with memory specificity, and fading affect bias—a greater reduction in the intensity of memory-related affect over time for negative versus positive events. We found that healthy participants demonstrated superior category fluency for positive relative to negative events but that this effect was absent in depressed participants. Healthy participants exhibited a strong fading affect bias that was significantly mitigated, although still present, in depression. Finally, memory specificity was reduced in depression for both positive and negative memories. Findings demonstrate that the positive bias associated with mental health is maintained by multiple autobiographical memory processes and that depression is as much a function of the absence of these positive biases as it is the presence of negative biases. Results provide important guidance for developing new treatments for improving mental health.

Good mental health is robustly associated with positive biases in varied aspects of self-perception (Taylor & Brown, 1988). Healthy individuals tend to rate positive attributes as more applicable to themselves than negative attributes (Brown, 1986). Their self-evaluations are consistently more positive than ratings made about them by independent observers (Lewinsohn, Mischel, Chaplin, & Barton, 1980) and more positive than the ratings they themselves make of other people (Alicke, Klotz, Breitenbecher, Yurak, & Vredenburg, 1995). Autobiographical memory (i.e., memories of personal life experiences) plays a fundamental role in shaping our sense of self, providing the basis from which we make judgments about self-worth and our place in the world (Conway & Pleydell-Pearce, 2000). As such, we have previously posited that the rose-tinted self-views associated with mental health are sustained by supporting processes within autobiographical memory and explicitly demonstrated a mechanism linking autobiographical memory processes to the overgeneralized positive self-views that characterize good mental health (see Hitchcock, Rees, & Dalgleish, 2017). This study sought to further evaluate the proposition that autobiographical memory processes are configured to support mental health by providing a comprehensive assessment of autobiographical memory processes that may be positively biased.

Existing literature provides evidence of bias toward positive information in a number of processes underlying the storage and retrieval of autobiographical memory. Positively valenced memories are retrieved more quickly (Lloyd & Lishman, 1975), with a greater level of sensory and contextual detail (D’Argembeau, Comblain, & Van der Linden, 2003), and with superior accuracy relative to negatively valenced memories (Matt, Vázquez, & Campbell, 1992). There is also superior preservation of emotion attached to positive memories relative to negative memories. The well-established Fading Affect Bias (FAB; Walker, Skowronski, & Thompson, 2003) refers to the fact that, in community samples, emotions associated with positive events typically decrease in intensity to a lesser extent over time than emotions associated with negative events. Walker and colleagues (2003) demonstrated that the strength of the FAB was closely related to emotional well-being, as an attenuation of the FAB was associated with increased levels of dysphoria (i.e., subclinical low mood). Overall, the literature demonstrates that in healthy samples, positively valenced autobiographical memories are both retained and retrieved more optimally than negatively valenced memories.

Although there is substantial evidence of increased detail, sensory, and emotional vividness of positive autobiographical memories in healthy samples, it has yet to be determined whether the *categorical fluency* of autobiographical memory recall is superior for the category of positively valenced memories.^1^ Tests of categorical fluency typically ask the examinee to generate as many exemplars of a given category (in this case “positive autobiographical memories”) as they can within a given time frame, following a prescribed set of rules (Thurstone, 1938).^2^ Success on category fluency tasks and other measures of so-called ideational fluency is therefore argued to require speeded productivity, divergent thinking, performance monitoring, executive control, and strategy generation (e.g., Vannorsdall, Maroof, Gordon, & Schretlen, 2012). Such tasks capitalize on both the degree of representational integration between category exemplars within the cognitive system, as well as the degree of association between exemplars and the overarching category or concept.

We therefore created a task designed to extend current research on category fluency into two new domains: autobiographical memory (i.e., memories of personal life experiences) and affective valence. This enabled us to examine, for the first time, autobiographical memory category fluency for positive and negative experiences. Elucidating the profile of personal memory category fluency across different domains of valence is important not only to extend our understanding of the nature and range of autobiographical memory effects relevant to mental health but also to establish a laboratory measure for future studies of memory category fluency and everyday cognition. For example, greater category fluency for positive events may underlie cognitive phenomena such as the “optimism bias” in healthy populations (Sharot, 2011) as a greater category fluency for related past events is likely to influence probability judgments regarding the likelihood of similar events occurring in the future (MacLeod & Campbell, 1992). Similarly, greater associative connections between positive information may underlie the positive (rather than neutral) mood state that is typical of healthy individuals and drives important evolutionary behaviors such as mating and socializing (Diener, Kanazawa, Suh, & Oishi, 2015). A task that indexes a variety of memory processes could therefore be used to elucidate the ways in which human cognitive systems may be configured to support a predominately positive mood and outlook on life, as well as subsequent adaptive behaviors. The first aim of the current study was therefore to determine whether the autobiographical memory processes are configured to allow more fluent retrieval of sets of memories within the positive valence category, relative to negatively valenced sets of memories, in healthy participants.

To assess this aim, we devised the Good Day–Bad Day (GD-BD) task, a paradigm based on methods proposed by Dritschel, Williams, Baddeley, and Nimmo-Smith (1992). The task requires participants to generate, separately, as many memories of positive or negative specific autobiographical events (i.e., events that lasted a day or less) as they can in a 2-min time window for each valence. Participants were asked to record a single word for each event that came to mind that would be sufficient to remind them of the event following completion of the task, for subsequent rating and coding. This approach maximized the number of events that could potentially be generated by minimizing the within-task information required per event. If there are underlying biases in favor of accessing and navigating positive memory stores (Conway & Pleydell-Pearce, 2000), we should expect greater category fluency (a higher number of generated memories within the time frame) for positive events relative to negative events in individuals with good mental health.

The second aim of our study was to investigate whether this hypothesized enhanced category fluency for positive autobiographical memories in healthy individuals was mitigated or even reversed in individuals with a diagnosis of depression. We know that depressed individuals recall greater numbers of negative past events in their life narratives compared to nondepressed controls (Fromholt, Larsen, & Larsen, 1995; Habermas, Ott, Schubert, Schneider, & Pate, 2008), and perceptions of self-identity and the personal future revolve around themes of worthlessness and failure (Gotlib & Joormann, 2010). Indeed, the idea that those with depression have a set of *negative* personal memories at their mental fingertips is central to cognitive models of the disorder and is proposed as a core mechanism that perpetuates depressive symptoms over time (Beck, 1967, 1976; Beck & Bredemeier, 2016). Laboratory research in which participants are directed to generate individual personal memories to sets of positive and negative cue words (Autobiographical Memory Test [AMT]; based on methods originally devised by Galton, 1883) also indicates that those with depression are faster to access individual negative personal memories to specific negative cues (Lloyd & Lishman, 1975), and diurnal increases in depressed mood accentuate this effect (Clark & Teasdale, 1982). However, although cued access to individual negative memories appears facilitated in depression, it is as yet unknown whether there is an elevated ability to generate sequences—*category fluency*—of related negative memories in those with the disorder, relative to positive memories (Dritschel et al., 1992).

Category fluency within autobiographical memory is likely to be critically implicated in the techniques used in cognitive-based therapies for mental health disorders such as depression. Cognitive therapy techniques (e.g., cognitive restructuring) require recipients to evaluate the validity of positive and negative ideas or beliefs about themselves by harvesting as many examples (i.e., pieces of evidence) as they can from autobiographical memory (and other sources) that are consistent or inconsistent with those ideas. These “evidence-gathering” techniques therefore depend heavily on autobiographical category fluency. If category fluency of positive information is impaired in depression, this would be reflected in a reduced number of individual pieces of information (e.g., “I got a High Distinction on my final university exam”; “I received good feedback on a piece of work last week”; “I completed a marathon last year”) that a depressed individual is able to provide when gathering evidence either to support a positive belief (e.g., “I am successful”) or to challenge a negative belief (e.g., “I am worthless”). As these therapeutic tasks are typically performed with a therapist within a number of minutes (Beck, Ward, Mendelson, Mock, & Erbaugh, 1961), developing adjunctive memory-based programs to improve category fluency, prior to the completion of cognitive therapy or similar interventions, might enhance treatment efficacy.

If there is greater integration between negative autobiographical memories in depression, we should expect greater category fluency (a higher number of generated memories) for negative events relative to positive events on the GD-BD task. On the other hand, it may be that autobiographical memory integration is “evenhanded” in depression with comparable category fluency for positive and negative events, reflecting findings in other domains of cognition associated with the disorder (e.g., depressive realism; Alloy & Abramson, 1979). In sum, our first study hypothesis was that there would be greater category fluency for positive memories relative to negative memories in healthy (never-depressed) participants, relative to either a reversed (negative memory bias) effect or an evenhanded category fluency profile in a depressed sample.

Another important aspect of the nature of recollected past negative events in depression is an apparent lack of specificity. In laboratory studies using the AMT, when those with depression are explicitly asked to retrieve specific autobiographical memories to word cues—memories of a particular event on a particular day—they instead show a greater tendency than healthy participants to retrieve categories of events that happened repeatedly or memories of events that extended over longer periods of time, with less contextual information and fewer specific details (Williams et al., 2007). For example, in response to the cue word *happy,* an individual with depression may say “when I was at school” as opposed to “I was happy at my 12th birthday party.” Meta-analysis of studies that have used the AMT demonstrate that such reduced specificity of recall in response to positive and negative word cues is a robust finding in depression (van Vreeswijk & de Wilde, 2004) and is not simply an epiphenomenon of the disorder. Reduced specificity appears to play a critical role in onset and maintenance, independently predicting the persistence of symptoms over time (Brittlebank, Scott, Mark, Williams, & Ferrier, 1993; Dalgleish, Spinks, Yiend, & Kuyken, 2001; Sumner, Griffith, & Mineka, 2010). Theoretical views propose that a reduced ability to access the specific details of the personal past underpins core maladaptive cognitive styles that characterize depression (e.g., rumination and avoidance), contributes to impaired problem solving and reduced ability to imagine future events (Jing, Madore, & Schacter, 2016; Williams et al., 1996), and contributes to overgeneralization of the negative self-beliefs that underlie depression (Hitchcock, Rees, et al., 2017).

Is it really the case that access to specific memories is impaired in depression? Although the discourse of depressed patients is characterized by negative categorical themes concerning the past (Beck, 1967), such narratives also contain an abundance of specific information about past difficulties (e.g., Ratcliffe, 2008), seemingly in contrast to laboratory findings using the AMT. One possible explanation for this apparent contradiction is that the phenomenon of reduced specificity may be an artifact of the AMT methodology. Rabbitt and Winthorpe (1988), on comparing cued to free recall of personal memories, found notable differences in the qualities of memories recalled using the two techniques, with cued memories tending to be less vivid, less emotional, and less spontaneously rehearsed. This indicates that the cue word method does impact how memories are recollected. One reason may be that the cues, as is the intention, constrain retrieval to cue-related content. When presented with cue words, depressed patients may actually recall a flood of specific, but cue-unrelated, negative memories, hindering their ability to retrieve specific memories that are actually related to the cue word.

By utilizing broad category cues as opposed to individual cues, the GD-BD task critically allows examination of autobiographical memory specificity independent of the constraints of individual cue words, thus allowing us to elucidate whether the memory bias associated with depression is an artifact of the AMT methodology. This is important given the centrality of this phenomenon to cognitive approaches to depression (Dalgleish & Werner-Seidler, 2014) and the nascent development of interventions for depression to address reduced specificity (Hitchcock, Werner-Seidler, Blackwell, & Dalgleish, 2017). Our second hypothesis was that a depressed group would generate a reduced proportion of specific memories on the GD-BD task, independent of valence, relative to healthy controls, in line with the extant memory specificity literature (Williams et al., 2007).

Finally, the GD-BD methodology provides the opportunity to examine further the concept of FAB. Walker and colleagues (2003) proposed that just as the FAB may support well-being in the mentally healthy, a modulated FAB might help explain the maintenance of depressed mood. However, to date, the FAB has not been examined in individuals with clinical depression, and it is thus unclear whether clinical levels of symptoms attenuate the effect, eliminate it altogether, or even reverse it. The final aim of the study was to evaluate the FAB effect in clinical depression. For each memory recalled on the GD-BD task, we asked participants to rate their emotions twice—once for their experienced affect at the original time of the recollected event and a second time then for their current levels of affect upon recollection of the event (cf. Clark & Teasdale, 1982). This allowed us to calculate an FAB index for each participant—the extent to which negative emotions to negative events decreased from the time of the event to the present more than did positive emotions to positive events. Our third hypothesis was that the FAB would be significantly attenuated, absent, or even reversed in depressed individuals relative to healthy (never-depressed) participants.

## Method

### Participants

Inclusion criteria were fluency in English and age over 18 years. Exclusion criteria comprised diagnosis of current substance dependence or a history of psychosis or organic brain injury. No participants were excluded on these bases. Participants with major depressive disorder (MDD; *n* = 21) were recruited via advertisements in local newspapers and health centers asking for volunteers to help with psychological research. MDD diagnosis and other current Axis I psychiatric comorbidity, according to the fourth edition of the *Diagnostic and Statistical Manual of Mental Disorders* (*DSM–IV;* American Psychiatric Association, 2000), were determined using the Structured Clinical Interview for the *DSM–IV* Disorders (SCID; First, Spitzer, Gibbons, & Williams, 1996). Nine participants had experienced too many previous major depressive episodes (MDEs) to distinguish the distinct number (as coded on the SCID), and for the remaining participants, there was a mean of 15 previous MDEs. Interrater reliability on the presence versus absence of diagnoses on the SCID for the current assessors was established for 21 participants, resulting in 100% agreement.

Healthy (never-depressed) participants (*n* = 23) reported no history of mental health difficulties, had no history of MDD according to the SCID, and scored no higher than the “mildly depressed” range (score < 19; Shaw, Vallis, & McCabe, 1985) on the Beck Depression Inventory (BDI; Beck et al., 1961), a well-validated measure of depressive symptoms.^3^ The “never-depressed” participants were recruited from the MRC Cognition and Brain Sciences Unit Volunteer Panel, a database of approximately 2000 community volunteers who have agreed to help with psychological research. Volunteers are recruited to the panel via advertisements in local newspapers.

### The GD-BD Task

The GD-BD task was broadly based on an autobiographical category fluency task described by Dritschel et al. (1992). In one component, participants were asked to remember as many good events as they could within 2 min. Written instructions were provided to participants, explaining that they should only remember good experiences from their past that were *specific*. Such experiences were defined as events that happened at a particular instance and/or lasted for a day or less and that made that particular day seem like a “good day.” Participants were instructed that their memories could be for events that had happened recently or a long time ago but must be from the participant’s own life. If a participant stopped generating events before the 2 min had elapsed, he or she was encouraged by the experimenter to continue.

In the second component, participants were asked to remember negative events, that is, “bad days,” and were given another 2 min to list as many specific negative experiences as they could, with the same parameters. The order of the two components (good days and bad days) was counterbalanced across participants. For each remembered event, participants were asked to write just one or two words, sufficient to later remind them of the event, and were instructed to avoid writing long descriptions of each event to conserve time. When the task had finished, participants were asked to use these short mnemonic cues to generate a fuller description of each memory. This enabled researchers to code the memories later (see below). Two examples were given to participants to clarify how to make notes during the task and how to summarize the events after the task. The dependent variables used to index memory category fluency were the number of good-day and bad-day memories generated.

#### Memory specificity

Each memory description was coded for specificity by a rater who was blind to condition and task instructions, according to the criteria described by Williams and Broadbent (1986). Specific memories were defined as events that happened in a particular instance and/or lasted for a day or less. Nonspecific memories included extended memories (events that lasted for longer periods of time) and categoric memories (events that occurred repeatedly). If participants retrieved the same specific memory more than once, offered responses that related to future events, or generated material that was not a memory (e.g., an opinion), a coding of “no memory” was given. Interrater agreement on a random selection of memories (*N* = 305) indicated good reliability, κ = .78.

#### FAB

After the GD-BD task was completed, and once participants had generated summaries of each memory recalled, they were asked to rate the retrieved memories in terms of both how happy/unhappy they had felt at the time the events occurred (THEN) and also how happy/unhappy they feel in the present (NOW) when they reflect back on the events, on a Likert scale from 1 = *extremely unhappy* to 7 = *extremely happy* (taken from Clark & Teasdale, 1982). Participants were also asked to state, as precisely as possible, when each memory occurred. To compute an index of FAB, emotion ratings for the bad-day memories were first reversed scored so that the scales for the two memory conditions were in the same direction and thus indexed emotional intensity independent of valence. We then subtracted these emotional intensity ratings for “NOW” from the emotional intensity ratings for “THEN,” separately for good-day and bad-day memories, and then subtracted this computed variable for good days from the computed variable for bad days to give an index of the FAB—the extent to which emotional intensity faded more for bad-day memories relative to good-day memories.

### Procedure

Ethics approval was granted by the Cambridge Psychology Research Ethics Committee. The study was conducted in a quiet testing room with the experimenter present at all times. During Session 1, participants provided informed consent, and the SCID was administered. In Session 2, approximately a week later, participants completed the GD-BD task, followed by the BDI. Finally, participants were debriefed and received a small honorarium (GBP£12) in acknowledgment of their contribution.

## Results

### Participant Characteristics

According to the SCID, in the depressed group, six participants also met *DSM–IV* criteria for current posttraumatic stress disorder, five for generalized anxiety disorder, three for specific phobia, three for social phobia, two for obsessive–compulsive disorder, one for panic disorder, one for hypochondriasis, one for agoraphobia, and one for bulimia nervosa. As expected, the depressed group (*M* = 25.05, *SD* = 7.23) scored higher on the BDI than the never-depressed group (*M* = 7.04, *SD* = 4.73), *t*(43) = 9.93, *p* < .001. Groups did not differ in gender, χ^2^(1) = 2.70, *p* = .10, or education history, χ^2^(4) = 3.26, *p* = .52. There was, however, a moderate-sized (but nonsignificant) effect for the never-depressed group (*M* = 40.04, *SD* = 16.36) to be younger than the depressed group (*M* = 47.36, *SD* = 12.95), *t*(41.57) = 1.67, *p* = .10. Given that age influences memory recall and category fluency (Kennedy, Mather, & Carstensen, 2004; Mathuranath et al., 2003), age was covaried in analyses.

### GD-BD Task Performance

Descriptive data for performance on the GD-BD task are presented in Table 1. Presented confidence intervals are 95% for Cohen’s *d* and 90% for η_p_^2^ (see Steiger, 2004). In terms of how long ago the remembered events took place (memory age), there was no significant difference between groups (good day: *F*(1, 41) = 0.26, *p* = .61, η_p_^2^ = .006 [.0, .09]; bad day: *F*(1, 41) = 1.93, *p* = .17, η_p_^2^ = .05 [.0, .18]) or between good-day memories compared to bad-day memories, *F* < 1, and no interaction between memory valence and group, *F* < 1. A manipulation check verified that the good-day memories were rated as markedly more positive at the time of their occurrence than the bad-day memories, *F*(1, 41) = 177.58, *p* < .001, η_p_^2^ = .81 [.71, .86]. There was also a main effect of group with the depressed group, as might be expected, rating memories at the time of their occurrence as less positive overall than did the never-depressed group across conditions, *F*(1, 41) = 4.66, *p* = .04, η_p_^2^ = .10 [.003, .25], but importantly, this effect was not significantly different as a function of memory condition, *F*(1, 41) = 1.50, *p* = .23, η_p_^2^ = .04 [.0, .16].[Table-anchor tbl1]

#### Memory category fluency

All analyses were initially performed with order (good-day task first vs. bad-day task first) as a factor, and there were no significant main effects or interactions involving this factor. Consequently, analyses without order are presented below.

**Figure 1 fig1:**
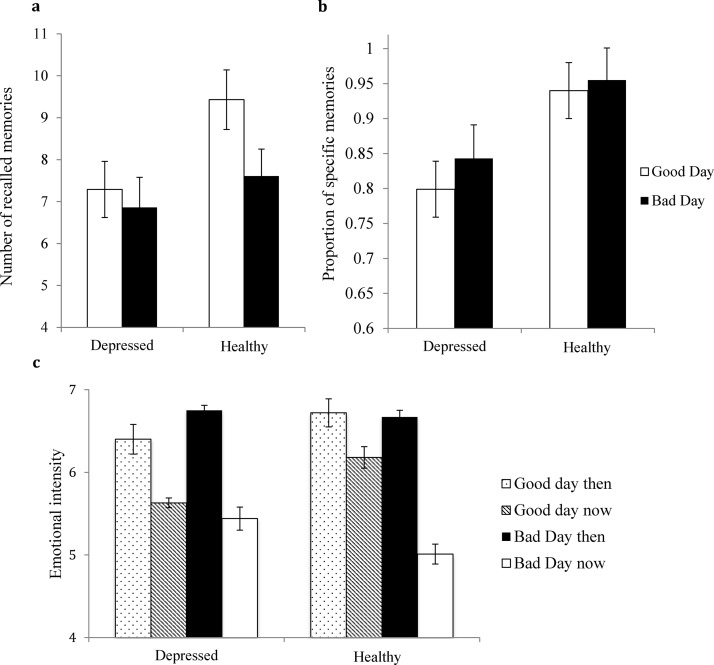
Mean (*SE*) category fluency, specificity, and fading affect characteristics for good-day and bad-day memories. (a) Total numbers of memories recalled. (b) Proportion of recalled memories that were specific memories. (c) Emotional intensity ratings at the time of the original event and at the present time.

We expected key differences between the never-depressed and depressed groups in their category fluency of positive and negative memory retrieval. Our first hypothesis was for a Group × Memory Condition interaction, such that the never-depressed group would experience more fluid retrieval of positive memories, demonstrated by a greater number of good-day memories than bad-day memories. We hypothesized this effect would not be evident in the depressed individuals, who would instead either recall a comparable number of good-day and bad-day memories or demonstrate a bias in favor of recalling more negative memories. We examined this hypothesis in a Group × Memory Condition analysis of covariance (ANCOVA; covarying for age) with the number of memories retrieved as the dependent variable (see Figure 1a). There were no significant main effects of memory condition, *F* < 1, or group, *F*(1, 41) = 2.73, *p* = .11, η_p_^2^ = .06 [.0, .20] (although the never-depressed group tended to recall more memories overall), but as predicted, we did observe a significant Memory Condition × Group interaction, *F*(1, 41) = 4.56, *p* = .04, η_p_^2^ = .10 [.003, .25]. As anticipated, the never-depressed group recalled significantly more good-day memories than bad-day memories, *t*(22) = 3.11, *p* = .01, *d* = 0.59 [−0.03, 1.21]. In contrast, for the depressed group, there was no significant difference in the total numbers of good-day and bad-day memories recalled, *t*(20) = 1.08, *p* = .29, *d* = 0.15 [−0.48, 0.78]. Our first hypothesis was therefore supported.[Fig-anchor fig1]

#### Memory specificity

The total numbers of specific, extended, categoric, and no memories are presented in Table 1. Proportions of specific good-day and bad-day memories, as a function of total number of actual memories recalled, are shown in Figure 1b. We used proportions to control for the aforementioned tendency for a difference between groups in the overall number of memories generated (Williams et al., 2007). A Memory Condition × Group ANCOVA revealed a significant main effect of group, *F*(1, 41) = 5.72, *p* = .02, η_p_^2^ = .12 [.01, .28], with the never-depressed group recalling a higher proportion of specific memories overall. There was no significant main effect of memory condition or a significant Memory Condition × Group interaction, *F*s < 1. As such, those with depression recalled a significantly lower proportion overall of specific memories than those with no history of depression, consistent with our second hypothesis.

#### FAB

Figure 1c demonstrates the emotional intensity ratings for memories at the time of the original event (THEN) and in the present (NOW) across conditions, with scores reversed for the bad-day memories to allow comparisons of intensity across condition. One-sample *t* tests with a reference value of zero demonstrated a significant FAB for both groups: never depressed, *M* = 1.14, *SD* = 0.75, *t*(21) = 7.11, *p* < .001, *d* = 2.14 [1.36, 2.92]; depressed, *M* = 0.53, *SD* = 0.69, *t*(20) = 3.50, *p* = .002, *d* = 1.08 [0.40, 1.76]. In line with our third hypothesis, a one-way ANCOVA with the FAB index as the dependent variable and age as a covariate revealed that the FAB index was significantly larger in the never-depressed group than in the depressed group, *F*(1, 40) = 6.97, *p* = .01, η_p_^2^ = .15 [.02, .31].^4^ These findings reveal that the FAB was relatively attenuated, but importantly not absent or reversed, in those with depression.

## Discussion

This study employed a novel paradigm, the GD-BD task, to compare the category fluency, specificity, and ratings of associated affect across time, for negative and positive memories in mentally healthy (never-depressed) versus depressed individuals. In line with our first hypothesis, the results revealed that healthy participants showed greater autobiographical category fluency—the ability to generate sequences of related memories—for positive compared to negative memories, whereas participants with depression showed comparable category fluency across positive and negative memory domains. To our knowledge, this is the first investigation of memory category fluency for events of different emotional valence, with findings suggesting that mental health is associated with more fluent recall of associated sets of positive relative to negative memories. Results also indicated that depression is associated with a relative reduction in the category fluency of positive memory retrieval rather than an overt category fluency bias in the domain of negative memories.

Evidence of a positive memory category fluency bias in healthy individuals reaffirms the importance of autobiographical memory processing in the maintenance of good mental health and extends our understanding of the mnemonic processes underpinning positivity effects to a novel cognitive domain (Vannorsdall et al., 2012). Future research establishing the mechanisms through which the evident memory biases directly impact well-being is now warranted. As noted earlier, we have previously demonstrated that autobiographical memory processes may be configured to support overly positive self-evaluations (Hitchcock, Rees, et al., 2017), and the current results suggest that biased memory category fluency may also contribute to broader self-serving mental biases and affective states (e.g., the optimism bias in evaluations about the future; Sharot, 2011), although this is in need of further investigation. Importantly, depression does not appear to be characterized by elevated negative autobiographical category fluency but rather by an even-handedness across valence domains. This is redolent of other effects in the memory literature, including recall biases (Blaney, 1986) and autobiographical memory specificity (see below; Williams et al., 2007). This supports the notion that, at the level of underlying cognitive processes, depression is as much a function of the absence of those positive self-serving biases that characterize mental health as it is of the presence of overt negative biases (Alloy & Abramson, 1979). In terms of therapeutic implications for depression, this indicates that efforts are better directed at enhancing category fluency of positive information through elaborating associations between positive memories and between memories and overarching positive themes, rather than focusing on correcting any negative category fluency biases.

In terms of specificity of recalled memories, in line with our second hypothesis, depressed participants recalled a significantly lower proportion of *both* positive and negative specific memories than never-depressed participants. This indicates for the first time that the reduced memory specificity effect robustly associated with depression in laboratory tasks using cued-recall methods (Williams et al., 2007) remains present when recall is minimally constrained as in the current GD-BD methodology. This is important because, if memory specificity effects were a confound of the cued-recall tasks that have typically been employed in specificity research, then the utility of interventions translated from this basic science literature would need to be reevaluated. Instead, evidence of reduced specificity across two methodological domains provides much more solid evidence for a depression-related deficit in the ability to recall specific memories, with clear implications for innovation of cognitive therapies (Hitchcock, Werner-Seidler, et al., 2017). Most notably, a critical challenge within cognitive therapies for depression is the requirement for recipients to collate specific information from their daily lives that is consistent or inconsistent with toxic self-related beliefs (e.g., “I am worthless”). The degree of specificity, concreteness, and detail of retrieved information will therefore have an impact on the efficacy of such techniques, and so any general differences in memory specificity are therefore likely to compromise the efficacy of cognitive therapy (Hitchcock et al., 2018). Recently, there has been concerted effort to develop low-intensity therapeutic interventions targeted at improving recall of specific autobiographical memories. These approaches have demonstrated beneficial impacts on both memory specificity and depressive symptoms (Werner-Seidler et al., 2018). The present findings corroborate this approach as worthwhile and suggest that completion of such programs prior to a course of cognitive therapy may mitigate this cognitive barrier to the success of therapy.

Our final set of findings showed that the FAB (Walker et al., 2003)—whereby affect associated with past negative events is experienced as reducing in intensity over time to a greater extent than affect experienced to positive events—was significantly attenuated in those with depression compared to healthy individuals who had never been depressed, although the effect was nevertheless present across both groups. The fact that the FAB was still present even in this clinically depressed sample perhaps testifies to the strength of underlying positive biases in affect-related processing that characterize the human cognitive system (Diener, Oishi, & Lucas, 2003). One thing that is important to note is that the FAB effect as measured here and in previous studies (Walker et al., 2003) is relying on retrospective ratings of past affect to past events. Consequently, the FAB effect is best conceptualized as a difference in the experienced fading of affect between positive and negative memories from the perspective of the present and is therefore potentially subject to mood-congruent memory effects. However, it is important to emphasize that the FAB is not an artifact of the task methodology, as the FAB is consistently observed in diary studies that have required individuals to rate emotional intensity of the event shortly after it occurs and then again upon recall of the event memory months or years later (e.g., Holmes, 1970; Walker, Vogl, & Thompson, 1997). It would be interesting to examine the FAB with contemporaneous affect ratings of past events in a clinically depressed sample. Similarly, further assessment of naturalistic retrieval styles, for example, using diary studies, may allow further examination of differences in retrieval between day-to-day and laboratory contexts.

In terms of future directions, the reduced specificity of memory retrieval has been demonstrated to persist into depressive remission, predisposing the individual toward future depressive episodes (e.g., Sumner et al., 2010). It would therefore be interesting to explore whether the attenuation of the FAB and reduction in positive memory category fluency are also evident in samples remitted from depression, as these processes may represent another facet of cognitive risk for depression, which could be targeted to enhance relapse prevention. Indeed, our findings indicate that the cognitive-based interventions to support emotional well-being are likely to be enhanced by adjunctive programs that aim to reinstate the positive autobiographical memory processes that characterize mental health.

## Context

This paper further elucidates how biases in autobiographical memory processes may maintain good mental health, and how alterations in the processing of autobiographical memories may impair emotional wellbeing. Findings from this study have implications for the development of autobiographical memory-based interventions which seek to promote emotional health; in particular, by demonstrating the need to focus on improving the specificity of recall, on enhancing category fluency of recall of positive material, and on techniques to maximize the positive affective benefit of positive memories.

## Figures and Tables

**Table 1 tbl1:** Mean (SD) Performance on the Good Day–Bad Day Task

Variable	Healthy		Depressed	
	Good day	Bad day	Good day	Bad day
Number of specific memories	9.04 (3.23)	7.26 (2.83)	5.95 (3.14)	5.86 (2.89)
Number of extended memories	.39 (1.03)	.30 (.56)	.67 (.91)	.71 (1.27)
Number of categoric memories	0 (0)	.04 (.21)	.67 (1.20)	.29 (.64)
Emotion ratings “THEN”	6.72 (.31)	1.33 (.36)	6.40 (.81)	1.25 (.26)
Emotion ratings “NOW”	6.18 (.61)	2.98 (.58)	5.63 (.77)	2.55 (.64)
Age of memories (months)	143.87 (117.06)	139.55 (118.81)	167.94 (128.93)	139.22 (105.73)
*Note*. Emotion ratings were on a Likert scale from 1 (*extremely unhappy*) to 7 (*extremely happy*).				

## Footnotes

1 It is important to distinguish category fluency from processing fluency (Alter & Oppenheimer, 2009), which refers to the ease of access to individual items of information (e.g., in the perceptual or memory domains) as opposed to *sequences of items* in the category fluency case.

2 Category fluency tasks are sometimes referred to as semantic fluency tasks when the categories are semantic (e.g., animals).

3 The original version of the BDI was used for legacy reasons within the department to enable longitudinal tracking of depression symptoms in participants.

4 There was a small effect size and no significant difference in the degree of fading affect for positive memories alone across groups, *F*(1, 41) = 1.31 *p* = .26, *d* = 0.35, but a moderate, although nonsignificant, effect size for negative memories alone, fading more in the healthy group than in the depressed group, *F*(1, 41) = 3.56, *p* = .066, *d* = 0.57.
